# Incorporating pathway information into boosting estimation of high-dimensional risk prediction models

**DOI:** 10.1186/1471-2105-10-18

**Published:** 2009-01-13

**Authors:** Harald Binder, Martin Schumacher

**Affiliations:** 1 Department of Medical Biometry and Statistics, University Medical Center Freiburg, Stefan-Meier-Str 26, 79104 Freiburg, Germany; 2 Freiburg Center for Data Analysis and Modeling, University of Freiburg, Eckerstr 1, 79104 Freiburg, Germany

## Abstract

**Background:**

There are several techniques for fitting risk prediction models to high-dimensional data, arising from microarrays. However, the biological knowledge about relations between genes is only rarely taken into account. One recent approach incorporates pathway information, available, e.g., from the KEGG database, by augmenting the penalty term in Lasso estimation for continuous response models.

**Results:**

As an alternative, we extend componentwise likelihood-based boosting techniques for incorporating pathway information into a larger number of model classes, such as generalized linear models and the Cox proportional hazards model for time-to-event data. In contrast to Lasso-like approaches, no further assumptions for explicitly specifying the penalty structure are needed, as pathway information is incorporated by adapting the penalties for single microarray features in the course of the boosting steps. This is shown to result in improved prediction performance when the coefficients of connected genes have opposite sign. The properties of the fitted models resulting from this approach are then investigated in two application examples with microarray survival data.

**Conclusion:**

The proposed approach results not only in improved prediction performance but also in structurally different model fits. Incorporating pathway information in the suggested way is therefore seen to be beneficial in several ways.

## Background

When using microarray data for analyzing connections between gene expression and a clinical response, such as survival time, additional knowledge is often available, e.g., on pathway or ontology relations. While several proposals exist, that take the latter into account, for statistical testing, there are only few techniques that consider such meta-information for building of predictive models.

One prominent source of knowledge on genes is the KEGG database [1]. Several authors have demonstrated that it can be highly beneficial to consider the pathway information found there into approaches for statistical testing [2-4]. While pathways can directly provide information on relations of genes, annotation databases, such as Gene Ontology [5], can also be employed for testing for the association between a clinical response and groups of genes (see [6], for example).

When building predictive models, Gene Ontology information, or the knowledge that two microarray features belong to the same pathway, can be incorporated by approaches that allow for explicit grouping of features [2,7]. Alternatively, pathway signatures can be developed. For example in [8], pathway signatures are determined by experimental techniques, and it is shown that these are related to survival in several independent cancer data sets.

However, simple grouping of features discards information on specific relations between genes within a pathway. A recent approach [9] not only uses the information that two genes are in the same pathway, but allows to incorporate information on specific gene relations. This is implemented by augmenting the log-likelihood criterion, to be maximized for estimating the parameters of a predictive model, by a penalty term that explicitly takes differences between the coefficients of linked genes into account.

As a basis for the approach in [9], the Lasso [10] is used, which provides for sparse estimates, i.e., predictive models where only few microarray features have non-zero influence. Similar to the fused Lasso [11], an additional term is added to the Lasso penalty. While there are techniques for fitting models to various response types when employing the original Lasso penalty [12], often only continuous response techniques are available for approaches which extend the Lasso penalty. Also, only an algorithm for estimation with a continuous response is provided for the approach in [9]. However, mainly binary and time-to-event responses are of interest for predictive microarray models.

Another problem with extensions of the Lasso approach is that several assumptions have to be made when choosing the structure of the penalty term. For example, the criterion employed in [9] penalizes the squared difference between (standardized) parameter estimates, which might be problematic when the true parameters have opposite sign. This is, e.g., the case when in a pair of connected genes one is up-regulated and the other one is down-regulated for patients with increased risk.

Boosting is an alternative technique for fitting high-dimensional predictive models (see, e.g., [13] for an overview). It uses a stepwise approach that allows to build up an overall model from many simple fits, refining the overall fit in every boosting step. When only the parameter estimate for one covariate is updated in each boosting step, componentwise boosting is obtained, resulting in sparse fits similar to the Lasso [14]. In addition, likelihood-based componentwise boosting allows for adequate consideration of clinical covariates in predictive microarray models [15,16]. The latter approach is available for all response types where estimation can be performed by Newton-Raphson steps for maximization of a likelihood, which are then adapted for penalized estimation in every boosting step.

For incorporating pathway information into boosting algorithms, one approach is to dedicate each single boosting step to the genes in one specific pathway [2]. However, just like grouping Lasso approaches, this does not take into account specific relations between genes.

As an alternative, we are going to adapt the componentwise likelihood-based boosting approach [15,16] for specifically incorporating pathway knowledge about gene relations into estimation of predictive models from gene expression data. The proposed *PathBoost *approach can be used for various response types, including binary and time-to-event responses. As pathway information is incorporated by adapting penalty parameters of connected genes in the course of the boosting steps, the approach also does not require an explicit specification of a penalty structure.

After outlining the details of the PathBoost algorithm in the following, it will be evaluated in a small simulation study, where it will be compared to the approach given in [9]. Its advantages on terms of prediction performance and interpretability are furthermore illustrated in two application examples with microarray survival data.

## Results and discussion

### The PathBoost algorithm

There are different response types for predictive models built from microarray data, the two most prominent being binary responses, employed, e.g., when classification of tumors is wanted, and time-to-event responses when prediction of survival is wanted. Our proposal for incorporating pathway information is based on likelihood-based boosting [15-17]. It is therefore suitable for all settings where parameter estimation can be performed by maximization of a likelihood via Newton-Raphson steps. For generalized linear models, the response, which might be continuous, binary or a counting response, is taken to be from an exponential family. Given observations (*y*_*i*_, *x*_*i*_), *i *= 1,..., *n*, with response *y*_*i *_and covariate vector *x*_*i *_= (*x*_*i*1_,..., *x*_*ip*_)', the structural part of such models is

*E*(*y*_*i*_|*x*_*i*_) = *h*(*η*_*i*_),

where *h *is a known link function and *η*_*i *_is the linear predictor

$$\eta_{i}=\beta_{inter}+{x^{\prime }}_{i}\beta ,$$

with intercept parameter *β*_*inter *_and parameter vector *β *= (*β*_1_,..., *β*_*p*_)', which are estimated by maximization of the log-likelihood *l*(*β*) (see e.g. [18] for more details).

In a time-to-event setting, observations (*t*_*i*_, *δ*_*i*_, *x*_*i*_), *i *= 1,..., *n*, typically comprise of an observed time *t*_*i*_, a censoring indicator *δ*_*i*_, that takes value 1 if the observed time is the time of the event of interest and value 0 if it is the time of censoring, and a covariate vector *x*_*i*_. Due to censoring, direct modeling of *t*_*i *_as a continuous response is problematic. Models for the hazard *λ*(*t*|*x*_*i*_), i.e., the instantaneous risk of having an event at time *t*, given the covariate information, are preferred.

The Cox proportional hazards model has the form

*λ*(*t*|*x*_*i*_) = *λ*_0_(*t*) exp(*η*_*i*_),

where *λ*_0_(*t*) is an unspecified baseline hazards and *η*_*i *_is a linear predictor of the form

$$\eta_{i}={x^{\prime }}_{i}\beta ,$$

with parameter vector *β*. Estimation of *β *is performed by maximizing the partial log-likelihood

$$l(\beta )=\sum_{i=1}^{n}\delta_{i}\left(\eta_{i}-\log\left(\sum_{k=1}^{n}I(t_{i}\leq t_{k})\exp(\eta_{k})\right)\right),$$

where *I*( ) is an indicator function that takes value 1 if its argument is true and value 0 otherwise, avoiding estimation of the baseline hazard.

#### Componentwise likelihood-based boosting

The basic idea of boosting is to fit several models to the data in a stepwise manner. In each boosting step, a new model is fitted, which gives larger weight to those observations that were fitted poorly in the previous boosting steps [19]. All individual fits are then combined into one overall model. It has been recognized that this procedure is in specific settings equivalent to gradient descent in function space [20], which in turn is equivalent to repeated fitting of residuals for the continuous response case with squared error loss function [14].

In [15], the latter idea is extended to generalized linear models by incorporating the previous boosting steps as an offset into the linear predictor *η*_*i*_. In [16], a similar approach for boosting estimation of the Cox proportional hazards model is suggested. The basic likelihood-based boosting algorithm is given in the following for both types of models.

Starting with parameter estimate ${\hat{\beta }}_{0}$ = (0,...,0), in each of *k*, *k *= 1,..., *M*, boosting steps, for each covariate *x*_*ij*_, *j *= 1,..., *p*, candidate models with linear predictor

$$\eta_{ij,k}={\hat{\eta }}_{i,k-1}+\gamma_{j,k}x_{ij}$$

are fitted by estimating parameters *γ*_*j*, *k*_. The offset ${\hat{\eta }}_{i,k-1}$ incorporates the information from the previous boosting steps, i.e.,

$${\hat{\eta }}_{i,k-1}={x^{\prime }}_{i}{\hat{\beta }}_{k-1}$$

for the Cox model and

$${\hat{\eta }}_{i,k-1}={\hat{\beta }}_{inter}+{x^{\prime }}_{i}{\hat{\beta }}_{k-1}$$

for generalized linear models. The intercept parameter ${\hat{\beta }}_{inter}$ is updated before each boosting step by fitting an intercept-only model.

For estimation of the *γ*_*j*, *k*_*s*, a penalized log-likelihood criterion

$$l_{pen}(\gamma_{j,k})=l(\gamma_{j,k})+\frac{1}{2}\lambda_{j,k}\gamma_{j,k}^{2}$$

is employed, where *λ*_*j*, *k *_is a penalty parameter that determines the size of the boosting steps. Typically, the same value of *λ*_*j*, *k *_= *λ *is employed for all covariates and all boosting steps. As the number of boosting steps *M*, which can, e.g., be determined by cross-validation, is the more important tuning parameter, the penalty parameter *λ *is chosen only very coarsely, such that the resulting number of boosting steps is not too small (say larger than 50).

Using score function *U*(*γ*) = ∂*l*(*γ*)/∂*γ *and information matrix *I*(*γ*) = -∂^2^*l*(*γ*)/∂^2^*γ*, more specifically the scalar values *U*_*j*, *k *_= *U*(0) and *I*_*j*, *k *_= *I*(0), we employ Newton-Raphson steps, resulting in estimates

$${\hat{\gamma }}_{j,k}=\frac{U_{j,k}}{I_{j,k}+\lambda_{j,k}}.$$

This is based on only one Newton-Raphson step, as further refinements can potentially be performed in later boosting steps.

The estimate ${\hat{\gamma }}_{j^{*},k}$ for the covariate with index j* which improves the fit the most (in terms of log-likelihood for generalized linear models or according to the penalized score statistic $U_{j,k}^{2}$/(*I*_*j*, *k *_+ *λ*_*j*, *k*_) for the Cox model) is then used to update the elements of the overall parameter vector via

$${\hat{\beta }}_{k,j}=\{\begin{array}{ll} {\hat{\beta }}_{k-1,j}+{\hat{\gamma }}_{j^{*},k} & \text{for }j=j^{*}\\ {\hat{\beta }}_{k-1,j} & \text{otherwise} \end{array}.$$

This componentwise boosting approach results in sparse fits, i.e., where many elements of the estimated parameter vector are equal to zero.

One of the advantages of likelihood-based boosting is that it is very easy to incorporate mandatory, unpenalized covariates (see [16], for example). This is useful when clinical covariates have to be incorporated in addition to microarray features, in order to compare the resulting model fit to a purely clinical model. The clinical covariates are then added to the linear predictor *η*_*i*_, and their coefficients are updated in or after every boosting step, but they do not enter into the penalty term.

#### Incorporating pathway information

The sparseness of the fits, resulting from approaches such as the Lasso or componentwise boosting, is a desirable property in settings with many microarray features, as it potentially results in a short list of genes, that are deemed influential. It can, however, also have a negative effect on interpretability. For example, if the level of activity of (parts of) a specific pathway is related to the response, the microarray features associated with that pathway will be highly correlated and have similar predictive power. However, sparse fitting techniques will probably pick out only one of the features. This makes it difficult to identify the underlying pathway. Also, model fits might be less stable when relying only on one measurement instead of several features.

For discouraging selection of only single microarray features associated with a pathway, we suggest to increase the penalty *λ*_j*, *l*_, *l *> *k*, used for a specific covariate *x*_ij*_, after it has been selected in boosting step *k*. This decreases the size of the boosting steps for this covariate and makes it less likely that this covariate will be selected in future boosting steps. In turn, the penalties for the microarray features that belong to genes that are directly connected in the respective pathway are decreased, making it more likely that they will be selected in future steps.

This approach requires specification of two rules, one for increasing the penalty of a selected covariate and one for decreasing the penalties for connected covariates. In the following, we provide such rules for penalty updates, which, in combination with componentwise likelihood-based boosting, constitute the *PathBoost *algorithm.

##### Increasing the penalty for a selected covariate

In order to provide a rule for penalty updates, a common metric for all covariates is needed. Therefore, we quantify the size of the boosting step *k*, performed for a covariate with index j* that has been selected in this step, by considering the estimate ${\hat{\gamma }}_{j^{*},k}$ relative to the estimate

$${\hat{\gamma }}_{j^{*},k}^{unpen}=U_{j^{*},k}/I_{j^{*},k}$$

obtained from unpenalized estimation, i.e., for *λ*_j*, *k *_= 0. The step-size factor *ν*_j*, *k *_then is given by

$$\nu_{j^{*},k}=\frac{{\hat{\gamma }}_{j^{*},k}}{{\hat{\gamma }}_{j^{*},k}^{unpen}}=\frac{I_{j^{*},k}}{I_{j^{*},k}+\lambda_{j^{*},k}}.$$

For incorporating pathway information, we suggest to decrease the step-size factor for a selected covariate by a constant step-size modification factor 0 <*c*_*smf *_≤ 1. So, after the covariate with index j* has been selected in boosting step *k*, the new step-size factor for further boosting steps *l *> *k *becomes

*ν*_j*, *l *_= *c*_*smf *_· *ν*_j*, *k*_,

implying a penalty increase via

(1) $$\lambda_{j^{*},l}=\left(\frac{1}{c_{smf}}-1\right)I_{j^{*},l}+\frac{\lambda_{j^{*},k}}{c_{smf}}.$$

For computational simplicity, we will use a fixed value of *I*_j*, *k*+1 _instead of the flexible term *I*_j*, *l *_in this penalty update rule. This means that the new penalty for a covariate can be calculated immediately after it has been selected in a boosting step and that the penalty stays the same until the covariate, or a covariate that is connected to it, is selected again.

##### Decreasing the penalty for connected covariates

If the penalty for a covariate *x*_ij* _is increased, and it is then selected again in a later boosting step, the explained variability due to this covariate and the pathways it belongs to will be decreased. To maintain the amount of variability explained by a pathway, the loss in explained variability for covariate *x*_ij* _is distributed to related covariates, e.g., to covariates that are connected to covariate *x*_ij* _in the pathway. The amount of potentially lost explained variability, that is to be distributed after a boosting step therefore has to be quantified. A proposal for this is provided in the following.

If *k *is the first boosting step where covariate *x*_ij* _is selected, then the unpenalized estimate ${\hat{\gamma }}_{j^{*},k}^{unpen}$ (obtained with *λ*_j*, *k *_= 0) will be approximately equal to the (unpenalized) maximum likelihood estimate ${\hat{\gamma }}_{j^{*}}^{ml}$ obtained from standard non-boosting estimation. As the relative step size, not realized due to penalized estimation, in boosting step *k *is given by 1 - *ν*_j*, *k*_, for boosting step *k *+ 1, the unpenalized estimate ${\hat{\gamma }}_{j^{*},k+1}^{unpen}$ will be approximately equal to

$${\hat{\gamma }}_{j^{*},k+1}^{unpen}\approx (1-\nu_{j^{*},k}){\hat{\gamma }}_{j^{*}}^{ml}.$$

Thus, the penalized estimate ${\hat{\gamma }}_{j^{*},k+1}$ will be

$${\hat{\gamma }}_{j^{*},k+1}=\nu_{j^{*},k+1}\cdot {\hat{\gamma }}_{j^{*},k+1}^{unpen}\approx \nu_{j^{*},k+1}(1-\nu_{j^{*},k}){\hat{\gamma }}_{j^{*}}^{ml}.$$

The approximate fraction *π*_*j*,(*m*) _of the maximum likelihood estimate that has been realized for covariate *x*_*ij *_in the *m*_*th *_boosting step, where this covariate has been selected, then is

$$\begin{array}{lll} \pi_{j,(1)} & = & \nu_{j,(1)}\\ \pi_{j,(2)} & = & \nu_{j,(1)}+(1-\nu_{j,(1)})\nu_{j,(2)}\\ & \cdots & \\ \pi_{j,(m)} & = & \pi_{j,(m-1)}+(1-\pi_{j,(m-1)})\nu_{j,(m)}. \end{array}$$

Let now *j*_1 _be the index of a covariate that has been selected in boosting step *k *and *j*_2 _be the index of the covariate to which a potential loss in explained variability is to be transferred. There is a potential loss that is incurred for $x_{ij_{1}}$ in a future boosting step *l *by employing a penalty that is updated via (1), with corresponding step-size factor $\nu_{j_{1},l}$, instead of not modifying the penalty, i.e., keeping the step-size factor $\nu_{j_{1},k}$. In terms of the fraction of the maximum likelihood estimate this loss is given by

$$(1-\pi_{j_{1},k})(\nu_{j_{1},k}-\nu_{j_{1},l}).$$

The aim is now to choose the penalty $\lambda_{j_{2},l}$, or correspondingly the step-size factor $\nu_{j_{2},l}$, for the covariate with index *j*_2 _for a future boosting step *l *(compared to step *k*), such that the loss for covariate $x_{ij_{1}}$ is compensated by covariate $x_{ij_{2}}$. Equating

(2) $$\left(1-\pi_{j_{2},k})\nu_{j_{2},l}-(1-\pi_{j_{2},k})\nu_{j_{2},k}=(1-\pi_{j_{1},k})(\nu_{j_{1},k}-\nu_{j_{1},l}\right)$$

results in an update for the step-size factor

$$\nu_{j_{2},l}=\nu_{j_{2},k}+\frac{1-\pi_{j_{1},k}}{1-\pi_{j_{2},k}}(\nu_{j_{1},k}-\nu_{j_{1},l}).$$

This implies a decrease of the penalty parameter $\lambda_{j_{2},k}$ via

(3) $$\lambda_{j_{2},l}=\frac{(1-\pi_{j_{2},k})I_{j_{2},l}}{(1-c_{smf})\frac{(1-\pi_{j_{1},k})I_{j_{1},l}}{I_{j_{1},l}+\lambda_{j_{1},k}}+\frac{(1-\pi_{j_{2},k})I_{j_{2},l}}{I_{j_{2},l}+\lambda_{j_{2},k}}}-I_{j_{2},l}.$$

Again, for computational simplicity, we use a fixed value of $I_{j_{1},k+1}$ instead of $I_{j_{1},l}$, and a value of $I_{j_{2},k+1}$ instead of $I_{j_{2},l}$ in this update rule. Therefore, the new penalties of connected covariates can be calculated immediately after the boosting step, avoiding recalculation after every boosting step and storage of results from past boosting steps.

As an increase of the penalty via (1) would leave the potential loss in explained variability undistributed for a covariate without connections, the penalty update is only performed for covariates that correspond to genes that have a connection to another gene, with corresponding covariate, in a pathway. For connected genes, however, the question remains whether the total amount should be transferred to every connected covariate or whether the right-hand side of (2) should be divided by the number of connections. As componentwise boosting results in very sparse fits, it can be expected that only few connected covariates will be selected in the remaining boosting steps. It therefore seems to be reasonable to assign the amount to each connected covariate.

While a measure of uncertainty is not available for connections in a pathway in the KEGG pathway database, it might be available from other sources. Such information could easily be incorporated into the PathBoost algorithm by multiplying the right-hand side of (2) by the measure of uncertainty (given that the latter has values between 0 an 1). Also, information on the direction of relations could be incorporated by propagating changes of a penalty only into one direction.

#### Choice of tuning parameters

The proposed PathBoost algorithm has three flexible parameters: an initial penalty *λ*_*j*,1 _= *λ*, *j *= 1,..., *p*, common to all covariates, the number of boosting steps *M*, and the step-size modification factor *c*_*smf*_. The initial penalty parameter is of minor importance and can be chosen very coarsely. A value that roughly corresponds to initial step-size factors of about 0.01 works very well in our experience. For determining the step-size modification factor *c*_*smf*_, a coarse line search is performed. For each value of *c*_*smf*_, the optimal number of boosting steps is determined by 10-fold cross-validation. Then the value of c_*smf*_, which results in the overall maximum of cross-validated (partial) log-likelihood, is chosen.

### Simulation study

To evaluate the performance of the PathBoost approach, we perform a small simulation study that is identical, in terms of design, to the study employed in [9]. Models for a continuous response are built from p = 2200 covariates. Of these, 200 take the role of transcription factors. The remaining 2000 covariates comprise of blocks of 10 covariates, where the covariates in each block are correlated with one specific transcription factor. The connection information, required for the approach given in [9] and for the PathBoost approach, is chosen such that there is a bidirectional connection between each transcription factor and each of the 10 covariates associated with it.

The true parameter vector in the generating linear model is chosen such that only four transcription factors (and the corresponding blocks of correlated covariates) have an effect on the response. There are six types of generating models with varying size and type of effect.

In Model 1, the true parameters of the covariates that are related to a transcription factor have the same sign as the parameter of the transcription factor itself. This is expected to be favorable for the approach given in [9], as the penalty term employed there penalizes the squared (standardized) differences of parameters. However, for true parameters with opposite sign, this difference will be large, making it rather unlikely that the true values are recovered. Model 2 features such a setting, where in each block of 10 informative covariates, the parameters of three covariates have a sign opposite to that of the associated transcription factor. In [9] it was found that this considerably affected the performance of the approach with an explicit penalty structure. In contrast, we do not expect a performance degradation for the PathBoost approach as it does not rely on differences of parameters.

Model 3 is similar to Model 1, and Model 4 is similar to Model 2, the only difference being a smaller effect of the covariates. Extending the design given in [9], we added two further settings, Model 5 and Model 6, which are based on Model 2 and Model 4 respectively. In these settings, only the first and the third block of informative covariates contain effects with opposite sign. Therefore, only six of a total of 40 informative connected covariates have an effect with a sign opposite to the associated transcription factor.

As a minimal performance reference, an intercept-only model, i.e., a model that does not use any covariate information, is fitted. A more specific performance reference for the PathBoost approach is provided by componentwise likelihood-based boosting without pathway information [15]. The main tuning parameter there is the number of boosting steps, which is determined by 10-fold cross-validation. As already suggested, the additional parameter *c*_*smf *_for the PathBoost approach is determined by a coarse line search.

As a performance reference for the approach given in [9], models are fitted by the Lasso [10], which also penalizes the absolute values of the parameters, but does not incorporate pathway information. For both approaches, fitting is performed by the least angle regression technique [21], which allows for fast computation of solutions for a large range of values for the penalty parameter that governs the absolute value term in the penalty. For the Lasso, only the latter has to be chosen, which is done by 10-fold cross-validation. For the approach given in [9], a second penalty parameter is required, which, similar to the PathBoost approach, is determined by a coarse line search.

All approaches are fitted to training sets of size *n *= 100, and prediction performance is evaluated on a test set of the same size. This is repeated 50 times. Table 1 shows the corresponding mean values and standard errors of the predictive mean squared error.

**Table 1:** Results of the simulation study.

Model	intercept	Lasso	Li&Li	lik.boost	PathBoost
1	762.5 (14.4)	83.6 (2.6)	42.5 (1.1)	83.4 (2.4)	61.0 (1.7)
2	305.8 (5.1)	91.0 (2.7)	80.8 (1.9)	89.7 (2.7)	64.8 (1.8)
3	215.6 (4.1)	32.6 (0.9)	24.9 (0.8)	32.1 (0.9)	26.5 (0.7)
4	131.1 (2.4)	32.6 (0.9)	29.9 (0.7)	32.5 (0.9)	26.9 (0.7)
5	525.7 (9.9)	87.9 (2.6)	61.6 (1.5)	85.6 (2.2)	62.2 (1.6)
6	171.6 (3.3)	32.9 (0.9)	27.6 (0.7)	32.2 (0.9)	26.9 (0.8)

Predictive mean squared error, mean and standard errors (in parentheses), for an intercept-only model, the Lasso, the pathway-based procedure proposed in [9] (Li&Li), componentwise likelihood-based boosting (lik.boost), and boosting with pathway information (PathBoost) for six types of generating models.

The predictive mean squared error for all approaches is far below that of the intercept-only model, indicating that the prediction problems are very simple. As would be expected, the performance of the Lasso and componentwise boosting is very similar. So, there is no disadvantage of choosing one of the two as a basis for an approach that incorporates pathway information.

The approach given in [9] outperforms the Lasso in all six settings. However, the performance difference is greatly diminished with Models 2 and 4, where several of the parameters of connected covariates have opposite sign. This highlights the difficulties potentially arising from an explicitly specified penalty structure. In contrast, the PathBoost approach is seen to result in a consistent improvement over boosting without pathway information in all settings. As would be expected from the design of the algorithm, the sign of the true parameters does not matter.

Comparing the PathBoost approach to that given in [9], the latter shows better prediction performance for Models 1 and 2, i.e., where its penalty structure matches the sign of the true parameters. However, for Models 3 and 4, where the sign of parameters of connected covariates may be different, the approach given in [9] performs worse. The performance of the two approaches is similar for Models 5 and 6, implying that already a small mismatch in sign information can nullify potential performance advantages gained by explicitly specifying the penalty structure in the approach given in [9].

### Application examples

In the following, we investigate the properties of the PathBoost approach in two application examples with microarray survival data, where a Cox proportional hazards model is fitted. When applying a technique for fitting predictive models that incorporates pathway information in a real application setting, there are two objectives. The first is to get better interpretability of the model fit, but the interpretation of a fit will only be credible if the second objective, that of improved prediction performance, is met. For adequately evaluating a potential gain in prediction performance from incorporating pathway information in a time-to-event setting, we employ bootstrap .632+ prediction error curve estimates [22-24].

Pathway information is extracted from the KEGG pathway database [1]. Similar to [9], we restrict analyses to regulatory pathways, but also include cancer pathways. As a restriction to gene-gene relations would have resulted in a very small number of connections, any genes that are linked by some kind of KEGG relation are considered to be connected.

While the glioblastoma data analyzed in [9] has a time-to-event response, closer inspection showed that the genes which have predictive power are not represented in KEGG pathways. Therefore, an approach focussed on the latter cannot improve over a null model that does not use any microarray information [25]. We investigate two other data sets, one from patients with large B-cell lymphoma [26] and a second from patients with ovarian cancer [8].

#### Diffuse large B-cell lymphoma

The data from patients with diffuse large B-cell lymphoma (DLBCL) has already been used for illustrating prediction error curve techniques [23] and the likelihood-based boosting technique for the Cox proportional hazards model [16], on which the PathBoost approach is based. Details of preprocessing are described there. There are *n *= 240 observation with *p *= 7399 microarray features. Only 1281 of the latter could be related to KEGG pathways, based on the information available. To avoid restriction to a (relatively) small number of microarray features and to maintain comparability to previous analyses, also the features not represented in KEGG pathways are considered.

A coarse line search, in combination with 10-fold cross validation, results in selection of a step-size modification factor of *c*_*smf *_= 0.9, which indicates that there might be some predictive pathway information in the data. Use of this factor results in 47 non-zero coefficients. In comparison, application of boosting without pathway information results in only 27 non-zero coefficients. There is an overlap of 20 non-zero coefficients, indicating that seven microarray features are no longer deemed important when pathway information is included, with 27 new features being added to the model.

For checking whether the 27 added features just contain information similar to the seven features not found in the PathBoost fit, we applied componentwise boosting to a data set where the seven features were removed. If the 27 seven features would be a substitute for the seven removed features, some of the former should now be included. However, the resulting model has 20 non-zero coefficients, which all belong to the same covariates as the overlapping coefficients above, i.e., none of the 27 microarray features, identified by PathBoost, are included in the model. The prediction performance decreases (not shown), indicating that the seven microarray features contain information which is useful in combination with componentwise boosting. However, as PathBoost does not utilize these seven microarray features and nevertheless performs better, this underlines that PathBoost results in structurally different model fits.

While in the model, fitted by boosting without pathway information, only two connected microarray features receive non-zero coefficient estimates, PathBoost results in 12 connected microarray features that receive non-zero estimates. This indicates that the fit from the latter algorithm reflects pathway knowledge. The coefficients of connected microarray features have different sign in several instances. As such a constellation did not influence the performance of PathBoost in the simulation study, an impact is also not expected in this application example.

**Figure 1:**
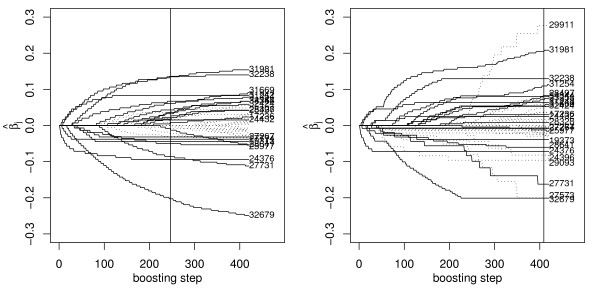
**Coefficient paths for the DLBCL data**. Coefficient paths for boosting without pathway information (left panel) and PathBoost (right panel), applied to DLBCL data. The models selected by 10-fold cross validation are indicated by vertical lines. Microarray features common to both models are indicated by solid curves, the others by dotted curves.

The change in structure of the fitted models is also seen from the coefficient paths, i.e., the parameter estimates plotted against the boosting steps. Figure 1 shows the coefficient paths for boosting without pathway information (left panel) and PathBoost (right panel). While they are rather similar, there are some features with strong effect that appear only in the PathBoost fit (e.g., UNIQIDs 29911 and 27573). As the PathBoost algorithm increases the penalty for a covariate after it has been selected, it could be expected that the estimates are somewhat shrunken compared to the CoxBoost fit. This is seen, e.g., for the microarray features with UNIQIDs 32238 and 32679, which are no longer selected by PathBoost after a certain boosting step, as the penalty for them has become too large. This is different from approaches that use an explicit shrinkage term in the penalized (partial) log-likelihood criterion, as there it would be expected that the whole path is shrunken.

**Figure 2:**
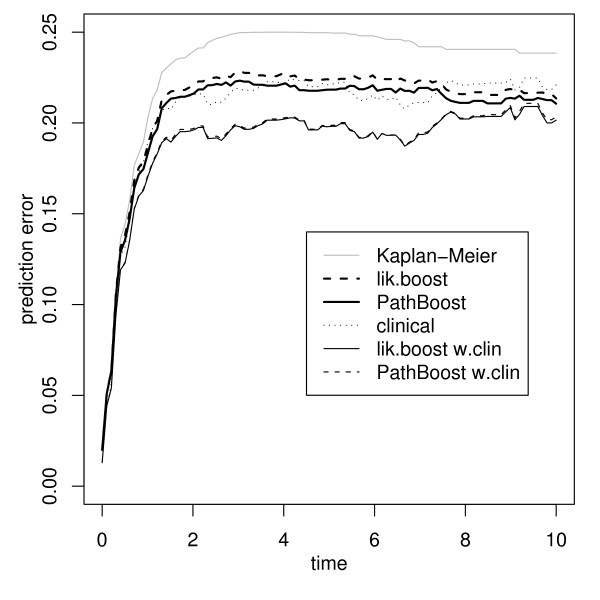
**Prediction error curves for the DLBCL data**. Bootstrap .632+ prediction error curve estimates for boosting without pathway information (dashed curves) and PathBoost (solid curves), applied to DLBCL data, without (thick curves) and with clinical covariates (thin curves). The Kaplan-Meier benchmark (grey curve) and a purely clinical model (dotted curve) are given as a reference.

While use of pathway information is seen to have influenced the model fit, interpretation of the latter can only be assumed to be more valid, compared to the fit obtained without pathway information, if prediction performance is also improved. The thick curves in Figure 2 indicate .632+ prediction error curve estimates (based on 100 bootstrap samples of size 0.632*n*, drawn without replacement). The Kaplan-Meier benchmark (grey curve) that does not use any covariate information is given as a reference. All procedures are seen to improve over the Kaplan-Meier benchmark, where PathBoost (solid curve) seems to have a slight advantage over boosting without pathway information (dashed curve). While the difference is not very large, it nevertheless improves the credibility of the PathBoost fit.

For 222 patients, a clinical predictor, the International Prognostic Index (IPI), is available. As it is typically of interest how much microarray information can improve over purely clinical models, we include the clinical covariate as a mandatory, unpenalized covariate, as described in [16]. The corresponding prediction error curve estimates are indicated by thin curves in Figure 2. The prediction performance of a purely clinical model is indicated by the dotted curve. It is seen that the combined models can improve over the purely clinical model. However, PathBoost (solid curve) can no longer improve over boosting without pathway information (dashed curve). The lack of additional value of pathway information in this setting is also reflected by the step-size modification factor, chosen by a line search, which is *c*_*smf *_= 1. Therefore it seems that, in the present example, pathway information is most useful in describing phenomena that are already reflected by the clinical covariate.

#### Ovarian cancer

The second data set, to be used for illustration of the PathBoost approach, is from patients with ovarian cancer. The original analysis of this data [8] already showed that there is a connection between pathway activity and survival, where pathway signatures were derived from prior experiments. In contrast, we will investigate whether pathway knowledge derived from the KEGG database can also add to prediction of survival.

For the 133 patients, where time-to-event information is available, we performed preprocessing of the microarray data, using the RMA approach [27], resulting in 21801 microarray features. We restrict analysis to those 4868 features that are related to any of the human KEGG pathways.

**Figure 3:**
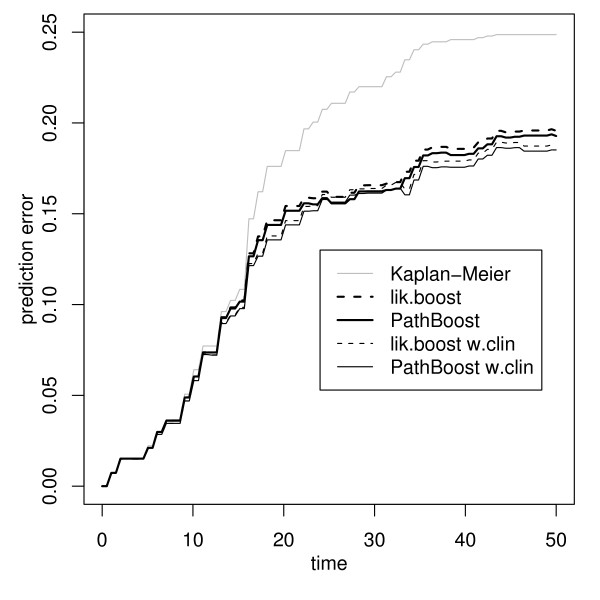
**Prediction error curves for the ovarian cancer data**. Bootstrap .632+ prediction error curve estimates for boosting without pathway information (dashed curves) and PathBoost (solid curves), applied to ovarian cancer data, without (thick curves) and with clinical covariates (thin curves). The Kaplan-Meier benchmark (grey curve) is given as a reference.

The connections between genes, just as for the DLBCL data, are extracted from the regulatory KEGG pathways, including the cancer pathways. The step-size modification factor, selected by a line search in combination with 10-fold cross-validation, then is *c*_*smf *_= 1, i.e., pathway information would not be expected to be useful for prediction of survival. However, when only the connections from the cancer pathways are considered, the resulting factor is *c*_*smf *_= 0.9. This indicates that targeted pathway information might be useful, while use of too many pathways is detrimental to prediction performance. Figure 3 shows bootstrap .632+ prediction error curve estimates for boosting without pathway information (thick dashed curve) and for PathBoost approach (thick solid curve), when considering only the cancer pathways. We also investigate models that incorporate the clinical covariate "tumor stage" as a mandatory unpenalized covariate (thin curves). All models perform considerably better than the Kaplan-Meier benchmark. Just as for the DLBCL data, there is an advantage of PathBoost over boosting without pathway information, albeit a smaller one, indicating usefulness of pathway information for prediction. In contrast to the DLBCL example, PathBoost also performs better when the clinical covariate is included. This indicates that the pathways provide information in addition to the clinical covariate.

## Conclusion

Integration of different sources of information promises to result in improved predictive models built from microarray data. For example, the potential of experimentally derived pathway signatures was already demonstrated in [8] for various independent cancer data sets.

Another source of pathway knowledge is the KEGG pathway database [1]. In [9], an approach was presented that utilizes this source for tailoring the penalty term in Lasso-like estimation. However, such approaches are not readily available for binary response and time-to-event data. Furthermore, they require explicit specification of a penalty structure, which is, e.g., problematic when the parameters of connected genes might have different sign.

As an alternative, we proposed a new likelihood-based boosting approach that also incorporates pathway information. Penalties are adapted after every boosting step, such that a microarray feature that is connected to another feature that already has a received a non-zero parameter estimate, is more likely to also receive a non-zero estimate. This avoids specification of a penalty structure, and therefore is not affected by parameters with opposite sign.

The proposed PathBoost was seen to perform well in various settings of a simulation study, using the design employed in [9]. While the approach given in [9] performed better in settings where the sign of the true parameters matched with its penalty structure, PathBoost showed equal or better performance in the other settings. This pattern of prediction performance might have been expected, as knowledge of the true sign of the parameters (in this case incorporated into the penalty structure) should result in increased prediction performance. However, in typical application settings such knowledge will rarely be available. Therefore, the PathBoost approach should be preferred. There still is a certain arbitrariness with respect to the suggested updated rules, i.e., other rules that might also work could be devised. However, the good performance, resulting from the suggested rules, provides at least some justification.

We employed the simulation design used in [9] to allow for better comparison to the results there. However, the design itself has some limitations, making it difficult to draw conclusions on performance with real data. For example, the pathway information employed does not contain inaccuracies, which will probably be present in sources such as the KEGG database. Also, the signal-to-noise ratios are large, resulting in simple prediction problems, untypical for microarray data. Furthermore, the simulation study is limited to continuous response settings, due to lack of an algorithm for the approach given in [9] for other response types. However, in most microarray applications the response is binary or a time-to-event response. Fitting predictive models for these is more difficult, and, therefore, less benefit from incorporating pathway information might be expected.

The proposed boosting approach is easily adapted to different response types. Variants for generalized linear models and the Cox proportional hazards model were given. The latter was employed in two application examples, where the gain in prediction performance by incorporating pathway information was more moderate, compared to the simulation study. As indicated, this might, e.g., be due to inaccuracies in the KEGG database. The estimated parameters of several connected microarray features had opposite sign, indicating similarity to those scenarios of the simulation study, where only PathBoost could fully utilize pathway information.

In comparison to models fitted without pathway information, application of PathBoost resulted in structurally different model fits, now honoring knowledge from external sources such as the KEGG database. Credibility of the interpretation of the new model fits was underlined by improved prediction performance. Given more detailed pathway knowledge, e.g., with information on the direction of gene relations and measures of uncertainty being available, further improvement of model fits could be expected. As demonstrated, the proposed boosting algorithm is highly flexible in terms of being able to incorporate additional sources of knowledge. While further refinements could be devised, e.g., for including information from Gene Ontology, it can already now be expected to provide for better model fits with better prediction performance in many applications.

## Authors' contributions

HB developed and implemented the initial version of the proposed algorithm, performed the simulation study, applied the algorithm to the example data, and wrote most of the manuscript. MS contributed design decisions for the algorithm, helped with interpretation of the results for the simulation study and the example data, and revised the manuscript.
